# A novel approach to measure brain-to-brain spatial and temporal alignment during positive empathy

**DOI:** 10.1038/s41598-022-18911-4

**Published:** 2022-10-14

**Authors:** J. Toppi, M. Siniatchkin, P. Vogel, C. M. Freitag, L. Astolfi, A. Ciaramidaro

**Affiliations:** 1 Department of Computer, Control and Management Engineering A. Ruberti, Sapienza University of Rome, Via Ariosto, 25, 00185 Rome, Italy; 2 IRCCS Fondazione Santa Lucia, Rome, Italy; 3 Department of Child and Adolescent Psychiatry and Psychotherapy, Protestant Hospital Bethel, University Clinics OWL, Campus Bethel, Bielefeld University, Bielefeld, Germany; 4 Department of Child and Adolescent Psychiatry, Psychosomatics and Psychotherapy, University Hospital Frankfurt, Goethe University, Frankfurt, Germany; 5 Institute of Neurophysiology, Neuroscience Center, Goethe University, Frankfurt, Germany; 6 Department of Biomedical, Metabolic and Neural Sciences, University of Modena and Reggio Emilia, Reggio Emilia, Italy

**Keywords:** Computational neuroscience, Social neuroscience

## Abstract

Empathy is defined as the ability to vicariously experience others’ suffering (vicarious pain) or feeling their joy (vicarious reward). While most neuroimaging studies have focused on vicarious pain and describe similar neural responses during the observed and the personal negative affective involvement, only initial evidence has been reported for the neural responses to others’ rewards and positive empathy. Here, we propose a novel approach, based on the simultaneous recording of multi-subject EEG signals and exploiting the wavelet coherence decomposition to measure the temporal alignment between ERPs in a dyad of interacting subjects. We used the Third-Party Punishment (TPP) paradigm to elicit the personal and vicarious experiences. During a positive experience, we observed the simultaneous presence in both agents of the Late Positive Potential (LPP), an ERP component related to emotion processing, as well as the existence of an inter-subject ERPs synchronization in the related time window. Moreover, the amplitude of the LPP synchronization was modulated by the presence of a human-agent. Finally, the localized brain circuits subtending the ERP-synchronization correspond to key-regions of personal and vicarious reward. Our findings suggest that the temporal and spatial ERPs alignment might be a novel and direct proxy measure of empathy.

## Introduction

Empathy is a basic component of social cognition, promoting effective interaction among subjects and motivating prosocial behavior. It can be defined as the ability to vicariously experience others’ feelings and emotions as a sort of alignment of emotional states among individuals including sharing others’ sufferings (negative empathy), but also feeling their joy (positive empathy)^1,2^. Studies on vicarious experience focused on the observation of others' pain or reward, hypothesizing the existence of a similar neural response to the observed and the same personal experience as signature of empathy^3^. However, whether empathy relies on overlapping processing of personal and vicarious experience is still widely debated^2^.

Neuroimaging studies reported overlapping activations during self- and vicarious experience in the subgenual anterior cingulate cortex (sgACC), anterior middle cingulate cortex (aMCC), temporal-parietal junction (TPJ), posterior superior temporal sulcus (pSTS), temporal pole, precuneus, medial prefrontal cortex (MPFC), supramarginal gyrus and dorsolateral prefrontal cortex (DLPFC)^4–8^. Specific additional activations during vicarious pain were found in the anterior insula and the anterior cingulate cortex^6^. Different neuroimaging studies suggest that individuals also share others’ positive emotional and bodily states during happiness and success^8–11^. However, positive empathy^11^ is a research topic that is still under development. Accordingly, the literature provides only initial evidence of the neural responses to others’ rewards, reporting specific activation in the ventral striatum^8^.

Similarly, EEG studies on vicarious experience also focused on comparable responses during the observation of others and during self-involvement. Moreover, they make it possible to exploit the temporal dynamics of event-related potentials (ERPs) to disentangle how the empathic response unfolds over time^12,13^. Three main ERP components were studied in relation to (positive or negative) vicarious experience. The medial-frontal negativity (MFN), a fronto-central component elicited between 200 and 300 ms following the stimulus, shows a higher amplitude in unfair/disadvantageous offers as compared to fair/advantageous ones during economic exchanges^14–16^. The P300 is a centro-parietal positive signal peaking between 300 and 600 ms following the stimulus. Several studies on vicarious experience during gambling tasks reported an increased P300 during gain as compared with loss, both in self and others^12,17,18^. Finally, the late positive potential (LPP) is a positive centro-parietal component occurring approximately 500–600 ms after stimulus onset. A modulation of the LPP amplitude was related to the sense of fairness modulated by social information during economic games, with more positive LPPs elicited by equitable offers than by inequitable ones^14,16,19^.

One paradigm particularly suitable to elicit a vicarious experience is the Third-Party Punishment (TPP^20^). In TPP, a subject, playing the role of dictator, is endowed with a sum of money, and must split it with another subject, the receiver, either in a fair (equitable) or in an unfair (unequitable) way. A third party not involved in the economic partition—called observer—observes the scene and may punish the dictator in case of a perceived unfair treatment of the receiver, at a personal cost (altruistic punishment). The paradigm is characterized by a phase in which receiver and observer are simultaneously notified of the dictator’s decision about the money sharing, and by a subsequent phase in which the observer is allowed to punish the unfair dictator’s behavior as a consequence of an empathic reaction. Similarly, when the dictator takes a fair decision, we can hypothesize that the observer positively empathizes with the receiver for the donation and vicariously perceives the reward. To date, TPP has only been used for the study of compassion and prosocial behavior in the context of social punishment^21–23^. However, shifting the focus to reactions to fair offers opens the way to the use of TPP also for the study of processes related to positive empathy and vicarious reward.

fMRI studies reported altruistic observer-related activation during unfair treatment in regions of the mentalizing network (MPFC and TPJ), in pain-related brain areas (anterior insula and anterior cingulate cortex) and in reward regions (ventral striatum)^24–29^. In ERP studies employing TPP, an increased modulation of MFN in the observer was associated with the violation of social expectancy and unequal offers^30–32^. Moreover, the ERPs during TPP showed the largest amplitudes of the LPP in the observer for fair offers over unfair ones^33^. ERPs thus appear to be modulated by the perceived fairness of a treatment received by others.

All the approaches adopted so far in fMRI and EEG-ERP studies on vicarious experience compared the “self” and “other” perspectives, thus evading the simultaneous interpersonal involvement of a natural vicarious experience.

To go beyond this perspective, a dual approach for the investigation of social functions was recently proposed: the so-called two-person neuroscience (2PN^34^). 2PN is based on hyperscanning, i.e., the simultaneous recording of brain signals of interacting agents, followed by the multivariate analysis of their neurophysiological signals^35^.

The first study employing EEG hyperscanning for TPP reported how vicarious pain can predict altruistic punishment^23^. More specifically, we demonstrated how specific indices characterizing multiple-brain connectivity between the receiver and the observer during the sharing of negative emotions can predict the subsequent observer’s prosocial behavior. The results confirmed the importance of simultaneously recording and analyzing the brain activity as well as the behavior of interacting subjects for depicting the specific relationship between them.

Hence, we extend our earlier results^23^ by proposing a new analysis to detect the event-related responses related to personal and vicarious reward as well as to analyze the synchronization of such responses, focusing on the positive empathy for the first time. This study aims to go beyond the “self-other” overlapping approach, toward the temporal alignment between ERPs in two individuals, which we hypothesize can represent the alignment of emotional states between them and therefore a measure of their ability to share emotions. Consequently, in this study, we use an EEG-hyperscanning setting to investigate synchronized brain responses between receiver and observer in a TPP experiment thus making it possible to capture the ecologic vicarious experience. Moreover, in order to modulate the participants’ response according to social information, we introduce the agency variable, splitting the role of dictator between a human and a non-human agent.

Our hypotheses are that (i) both the observer and the receiver present scalp-detected ERPs modulated by the fairness of the offer; (ii) there is a receiver–observer ERPs synchronization following the dictator’s money sharing; (iii) the amplitude of such synchronization is modulated by the agency and (iv) it is localized in key brain regions related to reward.

To test such hypotheses, we propose a novel approach. This is able to quantify the temporal synchronization between the ERPs in the dyad, based on a multi-subject computational model that links dynamical ERPs coherence with EEG source localization, aiming at identifying the specific brain regions underlying the fair treatment experienced by the receiver and vicariously shared by the observer. This study is novel from a neurocognitive point of view as well as from a methodological perspective. In fact, here for the first time we introduce the investigation of vicarious reward in a dual setting, describing the specific relationship between interacting subjects and going beyond the search for overlapping activity in the same subject during self- and vicarious experiences. Moreover, the approach here proposed—consisting of the analysis of coherence between ERPs in a dyad of interacting subjects—has never been applied before in the literature and thus provides a new tool, which will be available to future social neuroscience studies.

## Results

This hyperscanning study involved 42 healthy male volunteers, organized into 21 dyads that performed the TPP paradigm in the roles of receiver and observer, while the dictator was a confederate. EEG signals were recorded simultaneously from each dyad using 61 EEG channels for each subject. Further details on the participants, the experimental setting and the recordings are reported in the Methods section. Results about behavioral data can be found in Supplementary Information (text and Table S1) and in^23^.

### ERP characterization

EEG signals simultaneously recorded from each dyad were segmented in the time interval during which the two subjects learned the result of dictator’s decision. After preprocessing, data were averaged according to the stimulus onset across trials for each subject and then across subjects in order to extract the ERPs elicited by the paradigm.

**Figure 1:**
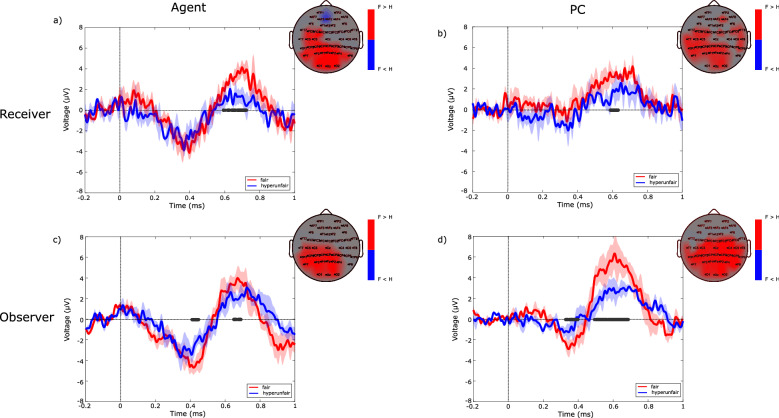
Grand Average (N = 14 dyads) waveforms at Pz location, obtained separately for receivers (**a**, **b**) and observers (**c**, **d**) according to different levels of fairness (fair in red and hyperunfair in blue), for the Agent (**a**, **c**) and PC (**b**, **d**) conditions. Each waveform is reported with its relative 95% confidence interval. The dark grey line over the x-axis indicates the interval for which the EEG potential is statistically different in fair and hyperunfair conditions (paired t-test, alpha = 0.05 validated by means of Guthrie–Buchwald method). Scalp maps reported in the upper-right part of each panel refer to the spatial distribution of LPP at peak latency. The maps are seen from above, with the nose pointing to the upper part of the page. Colormap codes for the t-values obtained comparing fair and hyperunfair condition within the group (paired t-test). Only statistically significant values are reported.

In Fig. 1 we reported the Grand Average waveforms obtained at Pz location, for receivers and observers, in Agent and PC conditions. A positive potential can be noted in the temporal window between 500 and 700 ms after the stimulus onset, for both receiver and observer in all conditions. However, receivers as well as observers exhibited significantly higher amplitudes of this potential in the fair compared to the hyperunfair condition. This was true for both agency conditions. Topographical maps obtained at the ERP peak (reported in the upper-right corner of each panel of Fig. 1) showed a parieto-occipital topography for such potential which is present in almost all the electrodes from the central line to the occipital one. Due to its temporal window and its centro-parietal topography we identified such potential as an LPP. The Grand Average obtained for the unfair condition was also checked, and it is reported in Fig. S1, together with the other two fairness conditions. No significant differences arose between the unfair and the hyperunfair conditions, therefore we decided to focus on the latter.

In Table 1 we reported the results of the ANOVA conducted separately on LPP amplitude and latency. We found only a significant effect of the factor FAIRNESS (F(1,25) = 44.59, p < 0.00001) on LPP amplitude, confirming the contribution of this factor to the LPP amplitude as revealed by Fig. 1. No significant effects resulted for the other factors (AGENCY and ROLE) on both LPP amplitude and latency.

**Table 1:** Results of the mixed ANOVA computed considering as within factors the agency (AGENCY: Agent, PC) and the fairness (FAIRNESS: fair, hyperunfair), as between factor the players’ role (ROLE: receiver, observer) and as dependent variables the LPP amplitude and latency, separately.

	d.o.f	LPP amplitude		LPP latency	
		F	p	F	p
ROLE	(1, 25)	0.419	0.523	0.642	0.430
AGENCY	(1, 25)	0.555	0.463	3.188	0.086
FAIRNESS	(1, 25)	**44.59**	**0.000001**	2.048	0.165
ROLE × AGENCY	(1, 25)	2.077	0.162	0.092	0.764
ROLE × FAIRNESS	(1, 25)	2.616	0.118	0.003	0.957
AGENCY × FAIRNESS	(1, 25)	0.034	0.855	0.792	0.382
ROLE × AGENCY × FAIRNESS	(1, 25)	1.038	0.318	0.152	0.699

Significant values are in bold.

### Receiver–observer LPP synchrony at scalp level

The temporal alignment between receiver’s and observer’s ERPs was quantified by means of a wavelet coherence analysis. A sample-by-sample paired t-test test was then used to assess if such alignment is modulated by the fairness of the dictator’s decision.

**Figure 2:**
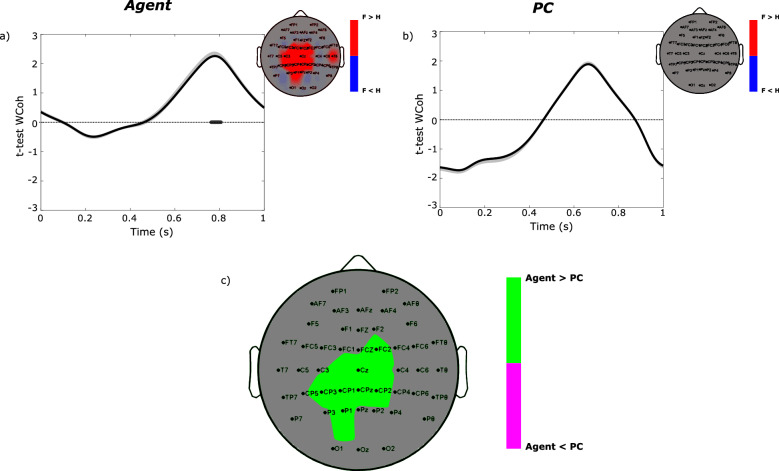
(**a**, **b**) Diagrams reporting the time course of t-values and the related 95% confidence interval obtained comparing the receiver–observer wavelet coherence in fair (F) and hyperunfair (H) conditions at Pz location, for the Agent (**a**) and PC (**b**) conditions. The dark grey line over the x-axis indicates the interval in which wavelet coherence is statistically different between fair and hyperunfair conditions (paired t-test, alpha = 0.05 validated by means of Guthrie–Buchwald method). Scalp maps reported in the upper-right part of each panel refer to the spatial distribution of wavelet coherence at the LPP peak. The maps are seen from above, with the nose pointing to the upper part of the page. Colormap codes for the significant t-values obtained comparing wavelet coherence in fair and hyperunfair conditions within the group (paired t-test). (**c**) Scalp map computed at LPP peak latency reporting the significant t-values obtained comparing the receiver–observer wavelet coherence in Agent and PC conditions (paired t-test, alpha = 0.05). We performed the comparison Agent (fair VS hyperunfair) VS PC (fair vs hyperunfair).

In panels a and b of Fig. 2 we reported the t-values obtained at Pz location by comparing wavelet coherence in fair and hyperunfair conditions, separately for Agent and PC. The ERP wavelet coherence for the fair and hyperunfair conditions, separately, can be found in Fig. S2. A significant fair-hyperunfair difference in the receiver–observer coherence resulted in correspondence of the time interval in which LPP reached its peak in single participants (around 700 ms). This means that the receiver–observer synchrony is higher in fair than in hyperunfair conditions, mainly around the LPP peak. The increase of t-values around LPP peak latency was found in both Agent and PC conditions, even if it reached significant values only in Agent condition. Topographical maps at LPP peak latency in Agent condition revealed that the receiver–observer coherence—significantly higher in fair than in hyperunfair conditions—is located over all the centro-parietal electrodes, in agreement with the well-known LPP topography. By comparing t-values obtained in agent and PC conditions at LPP peak (Fig. 2c), we found a higher receiver–observer synchrony when the dictator is a human agent than when it is a PC. Such difference is characterized by a centro-parietal topography as the one showed by LPP.

### Receiver–observer LPP synchrony at the source level

In order to evaluate the spatial alignment between receiver’s and observer’s ERPs, the wavelet analysis was conducted also in the source domain. EEG data were localized in 11 regions of interest (ROIs, see Table S2 in supplementary information) by means of eLORETA algorithm^36^.

**Figure 3:**
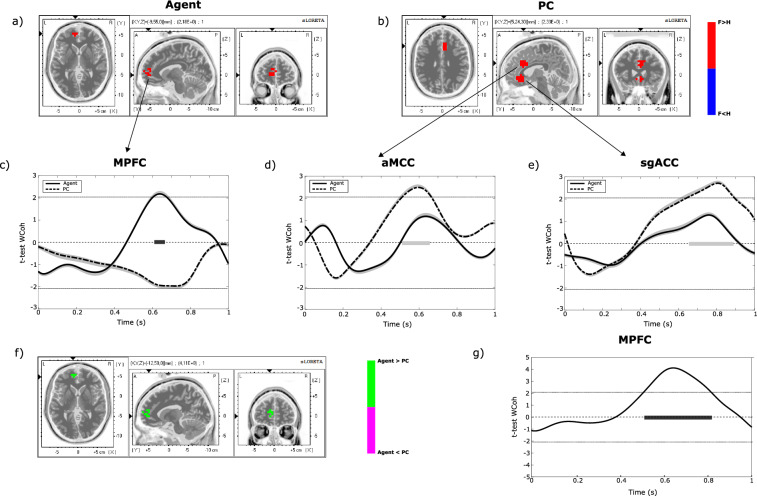
(**a**, **b**) Grand Average 3D statistical maps over LPP peak latency (600–700 ms). The colorbar represents the t-value obtained comparing receiver–observer wavelet coherence in fair and hyperunfair conditions (paired t-test, alpha = 0.05), in the Agent (**a**) and PC (**b**) conditions. Only statistically significant values are reported. (**c**–**e**) Diagrams reporting the t-values and the related confidence interval for Agent and PC conditions obtained at MPFC (**c**), aMCC (**d**) and sgACC (**e**) locations, comparing receiver–observer wavelet coherence in fair and hyperunfair conditions. The solid dark and light grey lines over the x-axis indicate the temporal interval in which the wavelet coherence is statistically different between fair and hyperunfair (paired t-test, alpha = 0.05, validated by means of Guthrie-Buchwald method) in Agent and PC conditions, respectively. (**f**) Grand Average 3D statistical maps over LPP peak latency (600–700 ms). The colormap represents the t-value obtained comparing the receiver–observer wavelet coherence in Agent and PC conditions (paired t-test, alpha = 0.05). Only statistically significant values are reported. (**g**) Diagram reporting t-values and the related confidence interval obtained at MPFC location comparing the receiver–observer wavelet coherence in Agent and PC conditions. The dark grey line over the x-axis indicates the temporal interval in which wavelet coherence is statistically different between Agent and PC conditions (paired t-test, alpha = 0.05, validated by means of Guthrie–Buchwald method).

The fair-hyperunfair comparison computed on receiver–observer wavelet coherence in each ROI revealed a significantly higher receiver–observer synchrony in fair than hyperunfair conditions over Medial Prefrontal Cortex (MPFC) when the dictator is an Agent (Fig. 3a) and over aMCC and sgACC areas when the dictator is played by a PC (Fig. 3b). Such differences were found in a time interval overlapped to the one in which we found the LPP, as highlighted by the fair-hyperunfair t-values reported along time for the three ROIs mentioned above (see panels c–e of Fig. 3). No differences in receiver–observer synchrony between fair and hyperunfair conditions were found for the other ROIs included in the study. The comparison of Agent and PC conditions revealed a higher receiver–observer synchrony in Agent than PC conditions over the MPFC area (Fig. 3f). Such difference is significant in the time interval of LPP peak latency (Fig. 3g).

## Discussion

Positive empathy supports prosociality and individual well-being^11^, but it has only rarely been studied in relation to social reward^8,37^. In the present paper we used TPP in an EEG hyperscanning setting to investigate ecological vicarious reward. To the best of our knowledge, this is the first study that employs the TPP paradigm for the study of vicarious reward, including a manipulation of the agency through human and nonhuman conditions. Moreover, here we proposed a new approach, suggesting that the temporal alignment between ERPs in the two individuals can be a measure of the ability to share emotions.

### Emotional sharing between receiver and observer

It was suggested that vicarious reward produces a sort of affective “co-experience” that includes sharing, celebrating and enjoying others’ positive emotions^11^. Accordingly, a first aim of this study was to investigate if the observer and the receiver presented similar scalp-detected ERPs modulated by the fairness of the offer. As hypothesized, for both roles and agency conditions, the grand average waveforms (see Fig. 1) showed the presence of a positive potential at a latency of around 600 ms, significantly higher in the fair with respect to the hyperunfair condition and with a centro-parietal topography, identifiable as the well-known Late Positive Potential.

Research on empathy showed that the LPP component reflects the intrinsic motivational relevance of the stimulus^38^ and seems to be involved during emotion regulation processes^39^. In fact, empathy is not a unitary concept^40^ and it is composed by two essential mechanisms: affective sharing and empathic concern. The LPP seems to reflect the latter, a top-down process during a vicarious experience. Other studies reported an association of LPP with higher cognitive functions related to the reward outcome as well as with the judgement of fairness based on social information^41^. Its amplitude modulation is larger in the fair compared to the hyperunfair condition (see Fig. 1a,b) and has already been described in previous works on economical exchange, such as the Ultimatum Game^14,16,19,42^. Furthermore, the presence of LPP in a third-party has been already highlighted in a previous work during fair offers as index of adherence to social norms^33^.

However, to the best of our knowledge, none of the studies published until now, simultaneously measured the person receiving the offer (a personal reward, in case of fair offer) and the one observing the social game, i.e., perceiving another person’s reward (vicarious reward). Moreover, it is important to note that the majority of studies investigating brain vicarious experience (positive and negative) adopt a single-subject design and use a conjunction analysis or an interaction analysis using self-emotion, no emotion and other’s emotion to identify neural responses related to vicarious experiences^2^ neglecting the social bond that characterized empathic experience. In this study, the use of a hyperscanning setting made it possible to study the presence of event-related potentials in two different subjects sharing an affective state, to compare their features (amplitude, latency, topography) and to describe the expected synchrony between the individual that personally experiences the reward (the receiver) and the individual (the observer) that vicariously experiences the positive emotion. However, this result can also be influenced by another factor: during the fair condition, the observed results are very consistent, i.e. the dictator makes a fair offer (10 points to him and 10 points to the observer) and the punisher has no reason to punish the dictator's behavior investing his points to sanction him. In contrast, during the hyperunfair condition, the interpretation of the dictator’s behavior by the receiver and the observer might not always be exactly the same or consistent across trials. In fact, the punisher’s behavior in this condition can vary from trial to trial. If the receiver and the observer have a different interpretation of the dictator’s behavior, this would result in a reduced brain synchronization. Therefore, we cannot exclude that the different degree of synchronization reported for fair and hyperunfair conditions could also be related to the heterogenous behavior that characterized the hyperunfair condition. However, as described above, since the literature supports the involvement of the LPP component during fair offers, we conclude that the different inter-subject synchronization is more likely to be due to a modulation of the reward outcome than to a different interpretation of unfair conditions.

Here we propose that the similarity of LLPs amplitude and latency might be a novel and direct proxy measure of empathy, since they indicate that the observer’s and receiver’s brain processes occur with the same magnitude and temporal scale, suggesting a temporal overlap between personal and vicarious experience. Moreover, by means of a time–frequency decomposition, we reconstructed the temporal evolution of the receiver–observer ERPs coherence, showing an increased point-to-point synchronization exactly in the LPP time window (see Fig. 2a,b). The receiver–observer synchronization is significantly higher for a fair compared to a hyperunfair treatment. The comparison with an appropriate condition is crucial for all hyperscanning studies, to discard any between-subjects synchronization due to the exposure to the same external stimuli or to the performance of the same task, and to reveal only the effects due to the cognitive processes at the basis of social interaction. Here these results suggest that such synchronization is modulated by the emotional content of the stimulus, as also reported by^33^. The LPP synchronization between both subjects can be seen as a true signature of emotional co-experience, which subtends the human inherent tendency to feel joy for others, even in the absence of personal economic gain, i.e. of vicarious reward^8^.

### Effect of the agency modulation on LPP synchronization

A second aim of this study was to investigate if the amplitude of ERPs synchronization was modulated by the agency. To the best of our knowledge, we were the first employing TPP for the study of vicarious experience including a manipulation of the agency through human and non-human conditions. We found higher LPP in fair than hyperunfair condition irrespectively of ROLE and AGENCY (Table 1), indicating that both personal and vicarious reward-related processes are not exclusively modulated by the presence of a human agent. Only few reward-related ERPs studies modulated agency, none of which addressing LPP. The results provided were controversial: some authors reported similar reactions to the human and computer conditions^15,43^, while others^12^ obtained ERPs only as a response to human agents’ behavior.

Moreover, we found an increase in LPP synchronization between receiver and observer as response to fair compared to hyperunfair offers in both agent and PC conditions (see Fig. 2a,b). However, such an increase is significant only when the dictator is a human agent, while in the PC condition there is only a trend in that direction, not confirmed by the statistical analysis. This was confirmed by the direct comparison between the two conditions (see Fig. 2c), which highlighted a higher synchronization in Agent vs PC, specifically in the LPP temporal interval and topological distribution. Our interpretation is that the increased LPP synchronization is due to the positive hedonic feeling caused by the kindness of a reward bestowed by a human stranger. In fact, this can be seen as a deliberated action driven by generosity and consequently carries more emotional significance to the recipient and the observer. In contrast, a fair offer made by the computer running on predetermined programs can be interpreted as a lucky random event. Some authors suggested that the LPP modulation induced by the social context might reflect the potential cognitive and attentional processing of the positive experience of valuable outcomes^33,41^ as generosity reasonably assumes more emotional significance than “blind” good fortune.

### Spatial and temporal alignment in key-regions of personal and vicarious reward

The literature describes a well-characterized neural network responsible for personal reward processing (for a meta-analysis paper see^44^) and some of these areas are also related to vicarious reward^2^. We sought to discover if the key regions of personal and vicarious reward subtend the inter-subject ERPs synchronization. Our wavelet coherence analysis showed a significantly higher receiver–observer synchrony during fair treatments over the MPFC when the dictator is an Agent (Fig. 3a) and over aMCC and sgACC areas when the dictator is a non-human agent (Fig. 3b). Moreover, the direct comparison revealed a higher receiver–observer synchrony in Agent than PC conditions over the MPFC area, again in the time interval of LPP peak latency (Fig. 3g). A recent meta-analysis identified a set of overlapping neural structures for personal and vicarious reward including the MPFC, the aMCC, the sgACC and other areas^45^. While our results are in line with the reported overlapping brain areas, here, for the first time, we additionally demonstrate that the overlap is not only “spatial” (same brain regions) but also “temporal” (same ERP temporally aligned). In fact, it is important to note that, being based on a simultaneous recording, the temporal and spatial alignment in reward key-regions reflects a real-time synchronized affective experience between two interacting people, a more functional alternative to undirected measurements using conjunction analysis and comparing a posteriori “self” with “other” conditions. Interestingly, we found a different spatial and temporal alignment in key-regions involved in personal and vicarious reward in accordance with the agency, i.e., MPFC when the dictator is a human agent and aMCC-sgACC when the dictator is a PC. Amodio and Frith in 2006 proposed a functional division of the medial frontal cortex (MFC): a posterior region of the rostral MFC—corresponding to our aMCC—implicated in action monitoring, reasoning about monetary gain and prediction error, an anterior region of the rostral MFC (corresponding to our MPFC) involved in self-knowledge and mentalizing, and an orbital region of the MPF (corresponding to our sgACC) associated with outcome. Following Amodio and Frith’s suggestions, we speculate that the spatial and temporal alignment showed in the MPFC subtends the self-thinking and mentalizing process that characterizes successful social interaction. It is notable that the activation of this region was reported during cooperative economic games exclusively when the participants believe to play a person rather than a computer^46,47^. Instead, the involvement of the aMCC and sgACC when the dictator is a non-human agent is probably explained by the feeling of a fortunate and unpredicted reward when the PC assigns a fair outcome, similarly to lottery winnings.

However, the neural mechanisms that underlie vicarious reward still represent a scarcely explored research area, and the literature still lacks studies reporting the manipulation of the agency factor during the simultaneous recording of individuals involved in positive affective experiences. Our work represents a first step in this direction, even if further studies are needed to confirm this explorative interpretation.

### Hyperscanning perspective in vicarious experience and ERP synchronization

In the present study, the hyperscanning approach not only allowed the detection of self-and vicarious reward by a comparison of the ERPs elicited in receiver and observer, but, more importantly, it made it possible to measure the (temporal) point-to-point synchrony between the ERPs simultaneously elicited in the two subjects, which was subsequently used to infer the spatial alignment between the brain activations. To this purpose, we adopted a tool already used in other contexts of EEG signal processing—the wavelet coherence—to quantify the sample-by-sample synchronization between the receiver and observer brain responses to the dictator’s decision. To the best of our knowledge, this instrument for tracking inter-individual synchronization is novel in the literature. EEG hyperscanning in ERPs paradigms was proposed for studying simultaneous ERP responses to visual or semantic stimuli^48–50^ without a multivariate analysis of inter-brain synchronization. Only one study measured the similarity between the ERPs of different subjects^51^ by computing the statistical differences in P300 amplitude and latency at single-trial level for two subjects playing the Prisoner’s dilemma. The novelty of our approach consists of computing—trial by trial—the synchronization between the two participants ERPs for the entire waveform, sample by sample, thus quantifying the true alignment between neural processes. Moreover, the detection of ERPs at single-trial level is maximized by means of a wavelet decomposition, and the synchronization is computed with a standardized estimator as the spectral coherence. As a result, our approach returns a thorough and statistically validated description of the spatial and temporal alignment between the two subjects’ brain activity.

Despite the novelty and potential value of this approach, some caution should be applied when using this procedure to correctly estimate the inter-subject ERP synchronization. First of all, it is well-known that the spectral coherence can be influenced by the power magnitude of the signals, e.g. for EEG epochs aligned according to a stimulus onset, where robust changes in power can be associated with simultaneous nonuniform phase angle distributions^52^. A check for the lack of correlation between the power magnitude of each ERP waveform and the phase of cross-spectra for the different time points is suggested to exclude this aspect. Secondly, as the frequency range of ERPS overlaps with the theta band, the wavelet coherence in the same range could as well be affected by concurrent changes in the theta activity. It is therefore mandatory to carefully relate the results of this analysis with the specific temporal alignment with a known ERP component and with the modulation of the function under investigation to draw significant conclusion.

Here, for the first time, we used an EEG hyperscanning setup to investigate vicarious reward. Previous studies focused on the reaction of an external observer to the reward felt by an individual and measured this vicarious experience in terms of activated areas, reconstructed via fMRI and often compared with the reaction to a personal win. The peculiar added value provided by hyperscanning is to depict the live interaction between individuals, going beyond the search for overlapping activity in the same subject during self- and other-experience. In fact, here we proposed a new approach quantifying the synchronization between ERPs elicited in two individuals as a measure of their shared positive emotion. We studied how the ERP synchronization is modulated by the fairness and by the dictator’s agency and subtended by specific key brain regions. It is interesting to note that despite the use of EEG recordings, we were able to associate such synchronization to specific brain regions involved in self and vicarious reward, as reported in previous fMRI studies. Besides providing new neurocognitive insights into vicarious reward, this work aims to enrich the social neuroscience panorama of methodological instruments potentially useful for future studies.

## Methods

### Participants

We included 21 pairs of right-handed male subjects, aged 18–30 years [mean 23.46 (SD = 3.7) and mean IQ 106.82 (SD = 13.33)] with normal or corrected-to-normal vision. Pairs were matched by age and IQ. Participants were recruited through advertisements in local schools and universities. All the procedures were carried out in accordance with the principles and guidelines of the Declaration of Helsinki, and all experimental protocols were approved by the local ethical committee of the Medical Faculty of Goethe University Frankfurt.

Psychiatric and neurological disorders were excluded by the Young Adult Self-Report (YASR)^53^ and a medical history interview. The YASR assesses emotional and behavioral problems in a standardized format regarding internalizing (such as anxiety, depression) and externalizing (such as hyperactivity, aggression) behaviors. The broad categories of internalizing and externalizing problems and total scores were used to exclude participants with symptoms of a mental or behavioral disorder (T-scores < 60). All participants were informed about the purpose of the experiment, and written informed consent was obtained prior to participation. A total of 15 out of 21 pairs [mean age 23.69 (SD 3.2) and mean IQ 108.75 (SD 13.6)] completed EEG recordings. The remaining 6 dyads were excluded from the subsequent analysis because of technical problems with the EEG recordings or low quality of EEG data for at least one of the two subjects. The subjects received a lump sum payment of 20 € for participation, in addition to the moneys they earned during the TPP game (range 0 to 30 €).

### Experimental setup

We implemented TPP with a third player within a classic Dictator Game^20^. The paradigm thus involves three players: the dictator (player A), the receiver (player B) and the observer (player C). Player A has an initial endowment of 20 points that he can share in different ways with player B, whose role is passive. In our study, we allowed three possible conditions: (i) 10 points to player A, 10 to player B (fair condition); (ii) 14 points to player A, 6 to player B (unfair condition); (iii) 18 points to player A, 2 to player B (hyperunfair condition). Player C observes the exchange and can use part or all his endowment (up to 4 points) to punish player A’s behavior. For every point invested by player C, 3 points are subtracted from player A’s payoff and 1 point is added to player B’ payoff.

In our study, we focused on the interaction between player B and player C (the receiver and the observer), whose EEG signals were simultaneously recorded in a hyperscanning setting. Our experimental subjects were randomly assigned to such roles, while the dictator role was played for half of the trials by the PC (PC condition) and for the other half by a confederate (Agent condition). The three subjects seated at the same table. Each dyad performed a total of 210 trials, divided into 7 blocks of 30 trials each, equally and randomly providing the three conditions (fair, unfair and hyperunfair). More details about the game and timeline of the trials can be found in^23^.

During the TPP paradigm, we collected the punishment score, assigned from the punisher to the dictator, in the various experimental conditions (more details could be found in supplementary information).

### EEG-hyperscanning recordings

Two parallel 64-channel EEG acquisition systems (Brain Product GmbH, Germany—for each subject: 61 EEG + 3EOG Ag/AgCl electrodes placed according to the 10–10 EEG system, referenced to linked mastoids, ground at Fpz) were used for the neuroelectrical hyperscanning recordings. The two systems were synchronized at hardware level by means of the Brain Vision USB 2 Adapter. The impedances were maintained below 10 kOhm. Sampling frequency was set to 250 Hz. EEG/EOG signals were filtered using a high-pass filter with a cutoff of 0.1 Hz. The sensitivity and the amplification gains were adjusted across the two amplifiers used for the recordings by means of a calibration signal. This made it possible to reduce the variance between the two amplifiers due to electrical noise and electrode impedance. Since the signals of two interacting subjects were recorded by a unique system, all problems related to the synchronization of the traces were mitigated.

### Pre-processing of EEG traces

EEG signals were band-pass-filtered in the range of 1–45 Hz. Independent component analysis (ICA) was used to remove ocular artifacts. In order to be as conservative as possible, we removed only one component per subject (the one identified as eye-blink artifact). For the ERP analysis, we considered the first 1000 ms after the presentation of the dictator’s decision to the other two players. We applied a baseline correction considering 200 ms interval preceding such window.

A semiautomatic procedure, based on a threshold (± 80 μV), was applied to remove residual muscular artifacts. Only epochs that were artifact-free for both subjects were considered in the subsequent analyses. No statistical differences between the experimental conditions were found in the number of clean epochs. A dyad was removed from the analysis due to many artifacts (in more than 50% of trials) in the EEG traces of one of the two participants. Consequently, the following analyses refer to data belonging to 14 dyads.

The pre-processing of EEG traces was performed by the software *Vision Analyzer 1.0* (Brain Product GmbH, Germany).

### Analysis of EEG data

#### ERP analysis in the receiver and the observer

To detect the ERPs elicited by the paradigm, EEG epochs were averaged according to the stimulus onset across trials. The procedure was repeated for each channel, each participant, each agency condition (Agent and PC) and for two levels of fairness (fair and hyperunfair). In particular, we focused on these two conditions since they refer to clearly characterized situations, i.e., an equitable and a strongly inequitable treatment. Then, from the averaged waveforms we extracted the amplitude and latency of ERP peaks in three channel locations (Fz, Cz, Pz) for each participant and subjected them to the statistical analysis described below. A grand average (across subjects) of the waveforms was then obtained separately for the two groups (receivers, observers) and for each agency and fairness level.

#### Observer–receiver ERPs synchrony: wavelet coherence

To measure the synchrony in ERPs elicitation between receivers and observers we used the wavelet coherence, a time-varying algorithm that made it possible to quantify the spectral coherence between two time-series, keeping the temporal information (see Supplementary materials for the details). We computed the wavelet coherence between the homologous time series (same electrode, same trial, same condition) which were simultaneously recorded in receiver–observer dyads. We used the Morlet function as the mother wavelet since it was previously indicated as appropriate for approximating the ERPs shape^54^ and we set the interval for the scale parameter in a range corresponding to the frequency range 4–7 Hz, typical of ERP components^55^. An average of the wavelet coherence parameters was then computed across trials for each couple and each experimental condition to arrange the data for the group statistical analysis (see paragraph 2.6.4). The wavelet coherence computation was implemented by means of wcoherence function in Matlab environment (*Matlab 2019b version*, Mathworks).

#### Receiver–observer ERPs synchrony in the source domain

In order to reconstruct the sources of EEG activity underlying ERPs we applied the exact Low-Resolution Tomography (eLORETA)^36^. EEG waveforms were subjected to the regularized linear inverse procedure aiming at projecting the scalp activities in the whole grey matter of the brain (see Supplementary materials). To accomplish the source localization, we used as solution of the forward model summarizing the propagation of the active sources to the EEG sensors, a lead field matrix extracted from the original New York Head model^56^. The New York Head is an accurate finite element electrical model of the average adult human based on a highly detailed nonlinear average of T1-weighted structural MR image of 152 adults provided by the International Consortium for Brain Mapping (ICBM). Moreover, it is composed of six tissue types (scalp, skull, cerebro-spinal fluid, gray matter, white matter, air cavities), instead of the three tissues of classical approaches. We used a 3D lead field matrix modeling the propagation of 5004 active sources spatially distributed in the whole grey matter towards 53 EEG sensors. We as a matter of fact had to remove eight EEG channels (TP9, O9, P11, IO, TP10, O10, Iz, P12) since they are not included in the New York Head model available online. The regularization parameter λ used for eLORETA solution was computed by means of a cross-validation approach^57^. The solution of the source localization problem was obtained for each subject and each experimental condition and consisted of a three-components waveform (one for each direction in the space) of the duration of one second per trial, for each of the 5004 dipoles used to model the grey matter. We then used the principal component analysis (PCA) to identify the dipoles orientation^58^. We applied the PCA to the source signal covariance matrix and then we selected the coefficients of the first principal component as versor of the dipoles^59^. Finally, we took the activity associated to the dipole closest to the centroid of each region of interest (ROI). In order to select independent ROIs, we selected 11 ROIs specifically related to positive and negative vicarious experience following^60^ and^45^. The list of ROIs and the related MNI coordinates can be found in supplementary materials (see Table S2 in supplementary information). The synchronization between homologous and simultaneous ROIs’ time series (same trial, same condition) in the receiver–observer dyad was computed by wavelet coherence, with the approach described in par. 4.5.2. The eLORETA solution was obtained by using the MATLAB-based Berlin Brain Connectivity Benchmark (BBCB)^61^, while wavelet coherence computation was implemented by means of *wcoherence* function in Matlab environment (Matlab 2019b version, Mathworks).

#### Statistical analysis

##### ANOVA on ERPs latency and amplitude

Values of ERP peaks latency and amplitude measured for each participant were included as separate dependent variables in two Mixed ANOVAs, computed considering as within factors AGENCY (two levels: Agent, PC) and FAIRNESS (two levels: fair, hyperunfair) and as between factor ROLE (two levels: receiver, observer). Tukey’s post-hoc test was used to identify differences among ANOVA factors. The analysis was performed using the software *STATISTICA* 8.0 (Stat Soft production).

##### Statistical analysis on ERPs waveforms

To assess the statistical differences between fair and hyperunfair EEG waveforms the Guthrie-Buchwald method was applied to explicitly take into account the dependences between successive time points in the ERPs waveform^62^. First we computed a dependent sample t-test (alpha = 0.05) between fair and hyperunfair conditions across subjects. We considered the sample-by-sample average EEG potential obtained across trials for each participant as a dependent variable, and as within factor the FAIRNESS (two levels: fair, hyperunfair). Then we identified segments of consecutive time samples resulted as significant by the t-test and we discarded all the segments composed by a number of samples below the empirical threshold provided by the Guthrie-Buchwald method. Considering an average first order auto-correlation of 0.9, a time window of 125 samples, 15 subjects and a graphical threshold of 0.05 we set the null-case threshold to 9 samples. The test was repeated for each channel, each agency condition (Agent, PC) and each group (receiver, observer). Since the two-way ANOVA on ERPs amplitude showed a significant effect only for FAIRNESS, we tested only the effect of such factor on ERPs waveform, separately for the two agency conditions. The analysis was executed using the statistical package in *Matlab 2019b* (Mathworks).

##### Statistical analysis on WCoh at scalp and source levels

Wavelet coherence waveforms obtained for fair and hyperunfair conditions as described in par. 2.6.2 and 2.6.3 were statistically compared across dyads by means of the Guthrie-Buchwald method (see the previous paragraph). The analysis was repeated for each sample in the observation interval. A t-value trend was extracted for each electrode and separately for the two agency conditions. The comparison between PC and Agent conditions was then performed by subtracting the t-values obtained for each of them, sample-by-sample. The analysis was repeated for each channel and the peak values were displayed over a map.

The statistical analysis above described was repeated for the wavelet coherence waveforms obtained for the ROIs in the source domain.

The analysis was performed using the statistical package in Matlab 2019b (*Mathworks*).

## Supplementary Information

Supplementary Information.

## Data Availability

The data in this study are available from the corresponding author on reasonable request.
