# Supplementary material for: A novel approach to measure brain-to-brain spatial and temporal alignment during positive empathy
Source: Sci Rep. 2022 Oct 14;12:17282. doi: 10.1038/s41598-022-18911-4 (PMC9568657; doi:10.1038/s41598-022-18911-4)
Supplement: Supplementary file 1 — Supplementary Information. [file 41598_2022_18911_MOESM1_ESM.docx]

**A novel approach to measure brain-to-brain spatial and temporal alignment during positive empathy**

J. Toppi^1,2^, M. Siniatchkin^3,4^, P. Vogel^4,5^, C. M. Freitag^4^, L. Astolfi*^$1,2^, A. Ciaramidaro^$4,6^

*^$^equally supervised this work*

*^1^Dept. of Computer, Control and Management Engineering A. Ruberti, Sapienza University of Rome, Rome, Italy*

*^2^IRCCS Fondazione Santa Lucia, Rome, Italy*

*^3^Department of Child and Adolescent Psychiatry and Psychotherapy, Protestant Hospital Bethel, University Clinics OWL, Campus Bethel, Bielefeld University, Bielefeld, Germany.*

*^4^Department of Child and Adolescent Psychiatry, Psychosomatics and Psychotherapy, University Hospital Frankfurt, Goethe University, Frankfurt, Germany*

*^5^Institute of Neurophysiology, Neuroscience Center, Goethe University Frankfurt/M, Germany*

*^6^Department of Education and Human Sciences, University of Modena and Reggio Emilia, Reggio Emilia, Italy*

**Supplementary Information**

**Analysis of Behavioral data**

We recorded the punishment score assigned by the observer to the dictator in all the experimental conditions. Such score was then subjected to a two-way repeated measures ANOVA, considering as main within factors the AGENCY (2 levels: Agent, PC) and the FAIRNESS (3 levels: fair, unfair, hyperunfair). The ANOVA was conducted on the 21 dyads enrolled in the study.

**Behavioral results**

Punishment score obtained as average across observer group is reported in Table S1 for the different experimental conditions. As expected, we found an increase of the punishment score across the three level of fairness, with hyperunfair condition showing the highest values (factor FAIRNESS: d.f. = (2.40), p < 0.00001, F = 92.44). Moreover, the punishment score was higher in Agent than in PC condition in both unfair conditions (interaction factor AGENCY X FAIRNESS, d.f. = (2.40), p = 0.019, F = 4.37).

|  | **fair** | **unfair** | **hyperunfair** | **significance** |
| --- | --- | --- | --- | --- |
| **Agent** | 0.3 (0.41) | 1.83 (0.73) | 2.93 (1.18) | F<U<HU |
| **PC** | 0.36 (0.46) | 1.64 (0.58) | 2.72 (1.14) | F<U<HU |
| **significance** | x | Ag>PC | Ag>PC |  |

*Table S1. Punishment score averaged across observer group reported separately for the different experimental conditions (fair(F), unfair (U), hyperunfair (HU) in both Agent (Ag) and PC (PC) conditions). Values in the brackets refers to standard deviation. The results of the ANOVA on punishment score highlighted an increase of the punishment score across the three level of fairness, with hyperunfair condition showing the highest values. The punishment score was higher in Agent than in PC condition in both unfair conditions.*

**List of the ROIs used in the EEG source localization**

| **ROIs name** | **ROIs acronym** | **MNI coordinates** | | |  |
| --- | --- | --- | --- | --- | --- |
|  |  | **x** | **y** | **z** | |
| Temporoparietal Junction | TPJ | 56 | -58 | 24 | |
| Right posterior Superior Temporal Sulcus | pSTS_R | 63 | -48 | 12 | |
| Left posterior Superior Temporal Sulcus | pSTS_L | -51 | -63 | 24 | |
| Temporal Pole | TP | 50 | 10 | -4 | |
| Precuneo | Prec | 12 | -44 | 6 | |
| medial Prefrontal Cortex | mPFC | -12 | 55 | 9 | |
| subgenual Anterior Cingulate Cortex | sgACC | 4 | 32 | -8 | |
| Supramarginal Gyrus | SMG | 48 | -44 | 29 | |
| Right Dorsolateral Prefrontal Cortex | DLPFC_R | 42 | 39 | 24 | |
| Left Dorsolateral Prefrontal Cortex | DLPFC_L | -51 | 39 | 24 | |
| anterior Middle Cingulate Cortex | aMCC | 6 | 18 | 30 | |

*Table S2. Names, acronyms and MNI coordinates of the ROIs used in this work. We specifically selected those related to positive and negative vicarious experience following*^1,2^*.*

**Wavelet Coherence**

Let’s consider two zero-mean random processes $x$ and $y$, the coherence between $x$ and $y$ at the frequency of interest $f$ is defined by

| $Coh(f)=\frac{\left\vert S_{xy}(f) \right\vert}{\left[ S_{xx}(f)\cdot S_{yy}(f) \right]^{1/2}}$ | (1) |
| --- | --- |

where $S_{xy}(f)$ is the cross-spectral density between the two processes $x$ and $y$ and it is computed as the Fourier transform of the cross-correlation function $R_{xy}(\tau)$:

| $S_{xy}\left( f \right)=\int_{-\infty}^{+\infty} R_{xy}(\tau)e^{-j2\pi f\tau}d\tau$ | (2) |
| --- | --- |

with

| $R_{xy}\left( \tau\right)=E[x\left( t \right)y(t-\tau)]$ | (3) |
| --- | --- |

where $E[]$ is the expectation operator.

When using Fourier analysis, as detailed above, the temporal information is lost by definition, since the spectra, and therefore coherence, are assumed to remain constant. However, for particular experimental situations where the same stimulation is delivered several times to a subject (as in event-related potential protocols), a variant of the temporal-smoothing coherence estimation has been proposed to regain temporal information. To overcome the problems due to non-stationarity, it has been proposed to apply wavelet analysis for the estimation of coherence among non-stationary signals. In contrast to Fourier analysis, wavelet analysis allows to analyze signals with rapidly changing spectra by performing a time-frequency analysis of the signal. Wavelet analysis is like the windowed short-term Fourier transform, but the size of the window is adapted to the frequency of the signal in wavelet analysis, and it is not fixed as in the short-term Fourier, resulting in a more accurate time-frequency resolution.

The wavelet transform of a signal $x(u)$ is a function of time $(u)$ and scale $s$ given by the convolution of $x$ with the wavelet family^3,4^:

| $W_{x}\left( u,s \right)=\int_{-\infty}^{+\infty} x\left( t \right)\frac{1}{\sqrt{s}}\Psi\left( \frac{t-u}{s} \right)dt$ | (4) |
| --- | --- |

where $u$ is the location parameter providing the exact position of the wavelet and $s$ is the scale dilatation parameter of the wavelet. In particular, $s$ defines how the wavelet is stretched or dilated, in fact, higher scale implies more stretched wavelet which is appropriate for detection of lower frequencies.

From the wavelet transforms $W_{x}\left( u,s \right)$ and $W_{y}\left( u,s \right)$ of two signals $x$ and $y$, we can define the wavelet cross-spectrum between $x$ and $y$ as follows^5^:

| $W_{xy}\left( u,s \right)=W_{x}\left( u,s \right)W_{y}^{*}\left( u,s \right)$ | (5) |
| --- | --- |

where $*$ denotes the complex conjugate, $u$ refers to the position index and $s$ is the scale.

The wavelet coherence is defined as the absolute value of the smoothed cross wavelet spectra normalized by the product of the smoothed individual wavelet power spectra of each selected time series. Formally, the squared wavelet coefficient is given as:

| $R^{2}\left( u,s \right)=\frac{\left\vert S(W_{xy}\left( u,s \right)) \right\vert^{2}}{S(\left\vert W_{x}\left( u,s \right) \right\vert^{2})S(\left\vert W_{y}\left( u,s \right) \right\vert^{2})}$ | (6) |
| --- | --- |

where $S$ denotes the smoothing parameter across both time and scale.

The squared wavelet coherence coefficient is in the range $0 \leq R^{2}\left( u,s \right) \leq1$ and values close to zero indicate weak correlation while that close to one confirms the presence of high correlation^6^.

## ERP characterization

| 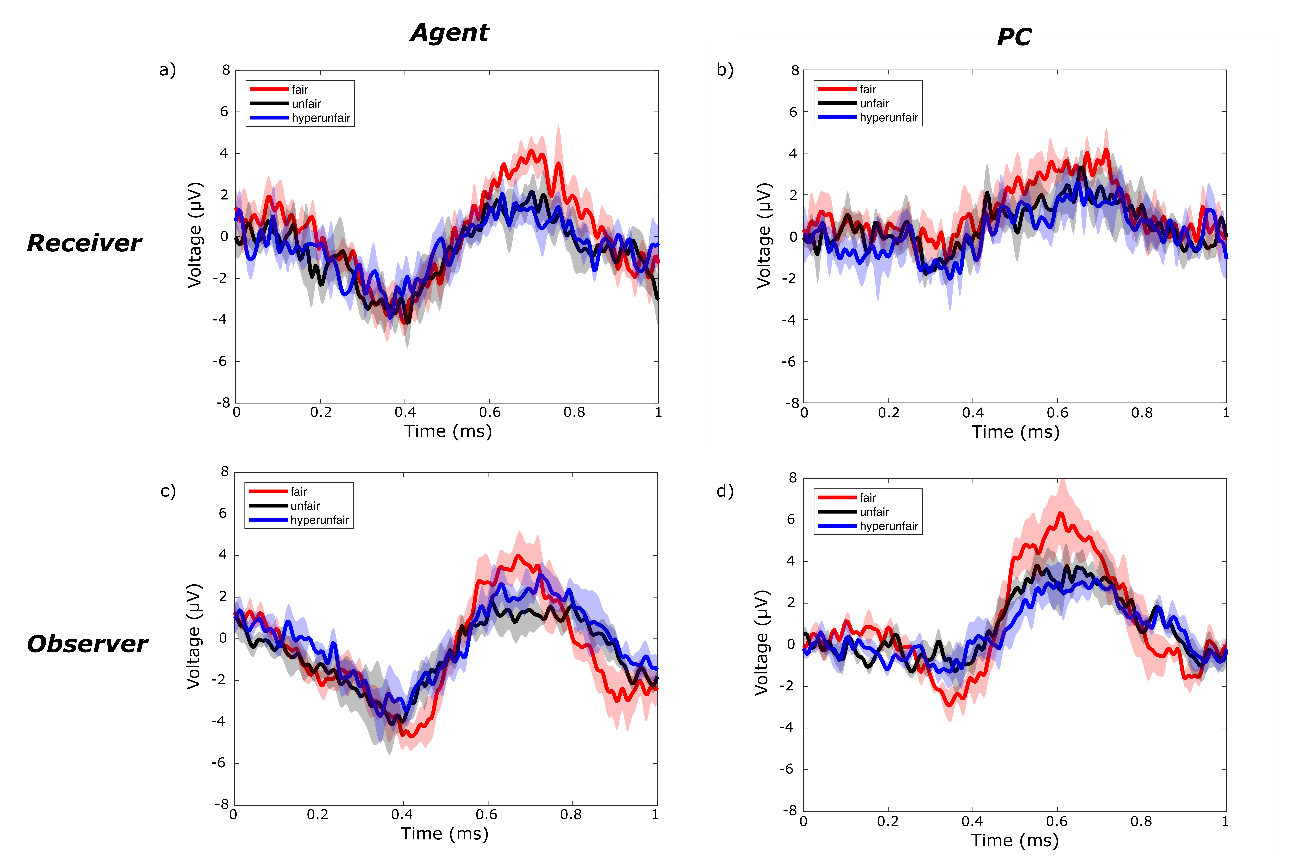 |
| --- |
| *Figure S1 – Grand Average (N=14 dyads) waveforms at Pz location, obtained separately for receivers (panels a and b) and observers (panels c and d) according to different levels of fairness (fair in red, unfair in black and hyperunfair in blue), for the Agent (panels a and c) and PC (panels b and d) conditions. Each waveform is reported with its relative 95% confidence interval.* |

In the main text of the manuscript, we focused only on the fair and hyperunfair conditions. In order to provide the reader with more details, in figure S1 we showed the same waveforms of figure 1 but we included also the ERP grand average related to unfair condition (in black in the figure). No differences were found between waveforms related to unfair and hyperunfair conditions for both receiver and observer in both Agent and PC conditions. For this reason, we decided to carry on all the following analysis excluding the unfair condition.

## Receiver-observer LPP synchrony at scalp level

| **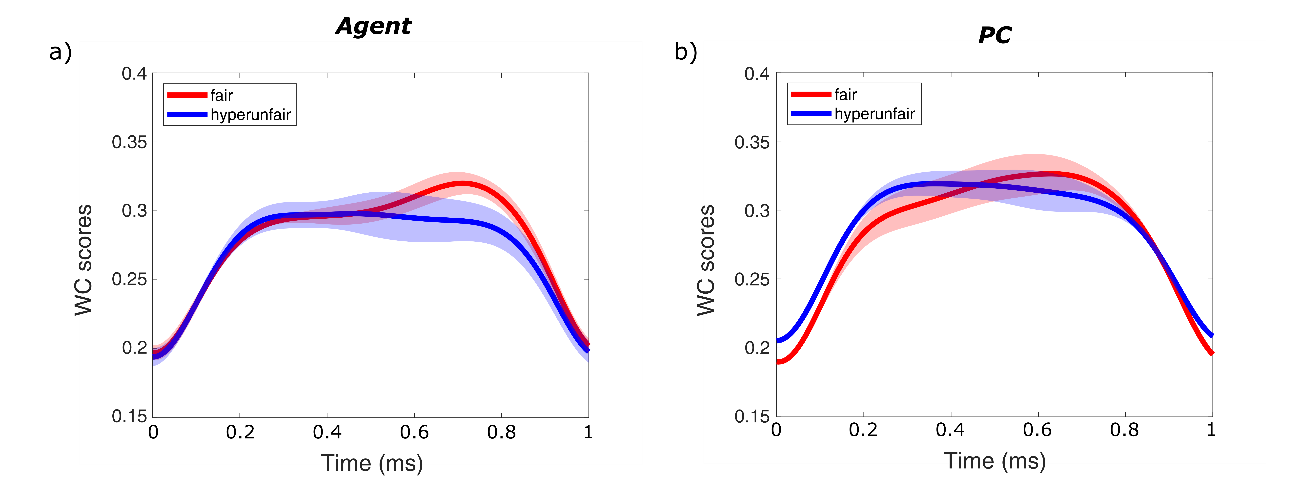** |
| --- |
| *Figure S2 – Grand Average of Wavelet Coherence between receiver-observer waveforms at Pz location for different levels of fairness (fair in red and hyperunfair in blue), for the Agent (panel a) and PC (panel b) conditions. Each waveform is reported with its relative 95% confidence interval.* |

**References**

1. Preckel, K., Kanske, P. & Singer, T. On the interaction of social affect and cognition: empathy, compassion and theory of mind. *Curr. Opin. Behav. Sci.* **19**, 1–6 (2018).

2. Morelli, S. A., Sacchet, M. D. & Zaki, J. Common and distinct neural correlates of personal and vicarious reward: A quantitative meta-analysis. *NeuroImage* **112**, 244–253 (2015).

3. Rua, A. & Nunes, L. C. International comovement of stock market returns: A wavelet analysis. *J. Empir. Finance* **16**, 632–639 (2009).

4. Vacha, L. & Barunik, J. Co-movement of energy commodities revisited: Evidence from wavelet coherence analysis. *Energy Econ.* **34**, 241–247 (2012).

5. Torrence, C. & Compo, G. P. A Practical Guide to Wavelet Analysis. *Bull. Am. Meteorol. Soc.* **79**, 61–78 (1998).

6. Lachaux, J.-P. *et al.* Estimating the time-course of coherence between single-trial brain signals: an introduction to wavelet coherence. *Neurophysiol. Clin. Clin. Neurophysiol.* **32**, 157–174 (2002).
